# Thyroid Nodule Management and Compliance With Guidelines in a District General Hospital in London: A Retrospective Study

**DOI:** 10.7759/cureus.84083

**Published:** 2025-05-14

**Authors:** Gideon Mlawa, Adnan Abdullah, Ayan Mohamed, Rabia Mahmood, Lina Eltayieb, Bashir Mahamud, Dalya Sadulah, Ehsan Shakoor, Godwin Simon, Ahmed Alh-Ghrairi, Furhana Hussein, Zahid Khan

**Affiliations:** 1 Internal Medicine and Diabetes and Endocrinology, Barking, Havering and Redbridge University Hospitals NHS Trust, Romford, GBR; 2 Internal Medicine, Barking, Havering and Redbridge University Hospitals NHS Trust, Romford, GBR; 3 Internal Medicine, Queen's Hospital, Romford, GBR; 4 Medicine, Barking, Havering and Redbridge University Hospitals NHS Trust, Romford, GBR; 5 Cardiology, University of South Wales, Pontypridd, GBR; 6 Cardiology, The University of Buckingham, London, GBR; 7 Cardiology, Barts Heart Centre, London, GBR

**Keywords:** american college of radiology (acr) thyroid imaging reporting and data system (ti-rads) criteria, benign and malignant thyroid nodule, british thyroid association, thyroid nodule, thyroid nodule and fine needle aspiration, thyroid nodule management, thyroid nodule size, thyroid nodule ultrasound, ti-rads 3, ultrasonography and thyroid cancer

## Abstract

Background: Thyroid nodules affect a small number of adults. Progress has been made to streamline and create a structured approach for evaluating and managing thyroid nodules, such as the use of ultrasound (US) U-scores (U1-U5) to stratify risk and guide the need for fine needle aspiration cytology (FNAC). This audit aimed to evaluate the adherence to the British Thyroid Association (BTA) 2014 guidelines in local practice at our local hospital.

Methods: This retrospective audit included 50 thyroid US reports over 12 months, identifying nodules and assessing compliance with BTA 2014 guidelines. Reports were evaluated for inclusion of a U-score or equivalent classification, such as the Thyroid Imaging Reporting and Data System score. Four patients did not have a U-score provided initially, which was retrospectively assigned by a consultant radiologist. FNAC performance was assessed based on U-score risk stratification: FNAC was expected for U3-U5 nodules, not U1 and U2. Cytology results were reviewed and classified using the THY system. The audit also analyzed reasons for deviation from guidelines by reviewing patients' medical notes for any documented reasons and the outcomes of histological analysis where surgical intervention occurred.

Results: Of the 50 US reports reviewed, 49 (98%) included a U-score. Distribution was as follows: U2 (13 cases), U3 (18 cases), U2/U3 (12 cases), U4 (five cases), and U3/U4 (one case). FNAC was indicated in 37 cases based on the U-score and was performed in 31. Despite being shown, six instances did not receive FNAC, often due to valid clinical reasons such as comorbidities, patient refusal, or alternative diagnoses. A total of 28 cases were classified cytologically using the THY system: THY 1 (one case), THY 2 (nine cases), THY 2c (one case), THY 3 (one case), THY 3A (seven cases), THY 3F (six cases), mixed THY 2/3 (one case), and THY 4 (two cases). Two cases had indeterminate THY 3 cytology, and one FNAC attempt was unsuccessful due to vascularity. Among the 31 cases assessed via FNAC, five were diagnosed with malignancy; four underwent hemithyroidectomy, and one had a local excision. Additionally, three patients who had hemithyroidectomy were found to have benign adenomas, most commonly follicular adenomas. Overall, 80% of cases adhered to BTA guidelines regarding FNAC indication. All U2 and U4 cases were managed in accordance with guidelines.

Conclusion: This audit demonstrates high compliance with the BTA 2014 guidelines, with near-universal documentation of U-scores and appropriate use of FNAC in most cases. While deviations were minimal and often clinically justified, there remains scope to improve standardization and adherence through targeted education and resource support. Continued emphasis on guideline-driven practice is essential to ensure optimal management of thyroid nodules and efficient use of clinical resources.

## Introduction

Thyroid nodules are frequently encountered, and about 50% of the population may have them. However, physically palpable nodules are noted in about 5%-7% of adults [1,2]. The incidence rate of thyroid cancer in the United Kingdom is estimated to increase by 74% between 2014 and 2035, to about 11 cases per 100,000 population by 2035 from its current level [1]. Thyroid cancer is the 17th most common malignancy among men and sixth among women [2]. However, autopsy findings indicate that nodules exceeding 1 cm are present in nearly 50% of individuals who had no prior diagnosis of thyroid disease [3]. The 2014 British Thyroid Association (BTA) guidelines suggest using a U-score to classify thyroid nodules according to their ultrasound (US) features. Based on their assigned U-score, they also recommend which nodules should undergo US-guided fine needle aspiration cytology (FNAC). Based on their cytology results, the guideline also recommended which sampled nodules require further management, including repeat FNAC (Thy-score). This has implications regarding time, cost, and the two-week wait pathway for thyroid Nodules [4]. The guidelines for thyroid nodule assessment may vary based on geographic location. For example, the Bethesda System for Reporting Thyroid Cytopathology is used in the United States to classify fine needle aspiration [5,6]. This audit aims to assess whether thyroid US reports have included a U-score for nodule classification and whether FNAC was performed appropriately based on the U-score in accordance with the BTA 2014 guidelines [7].

## Materials and methods

Methodology

**Figure 1:**
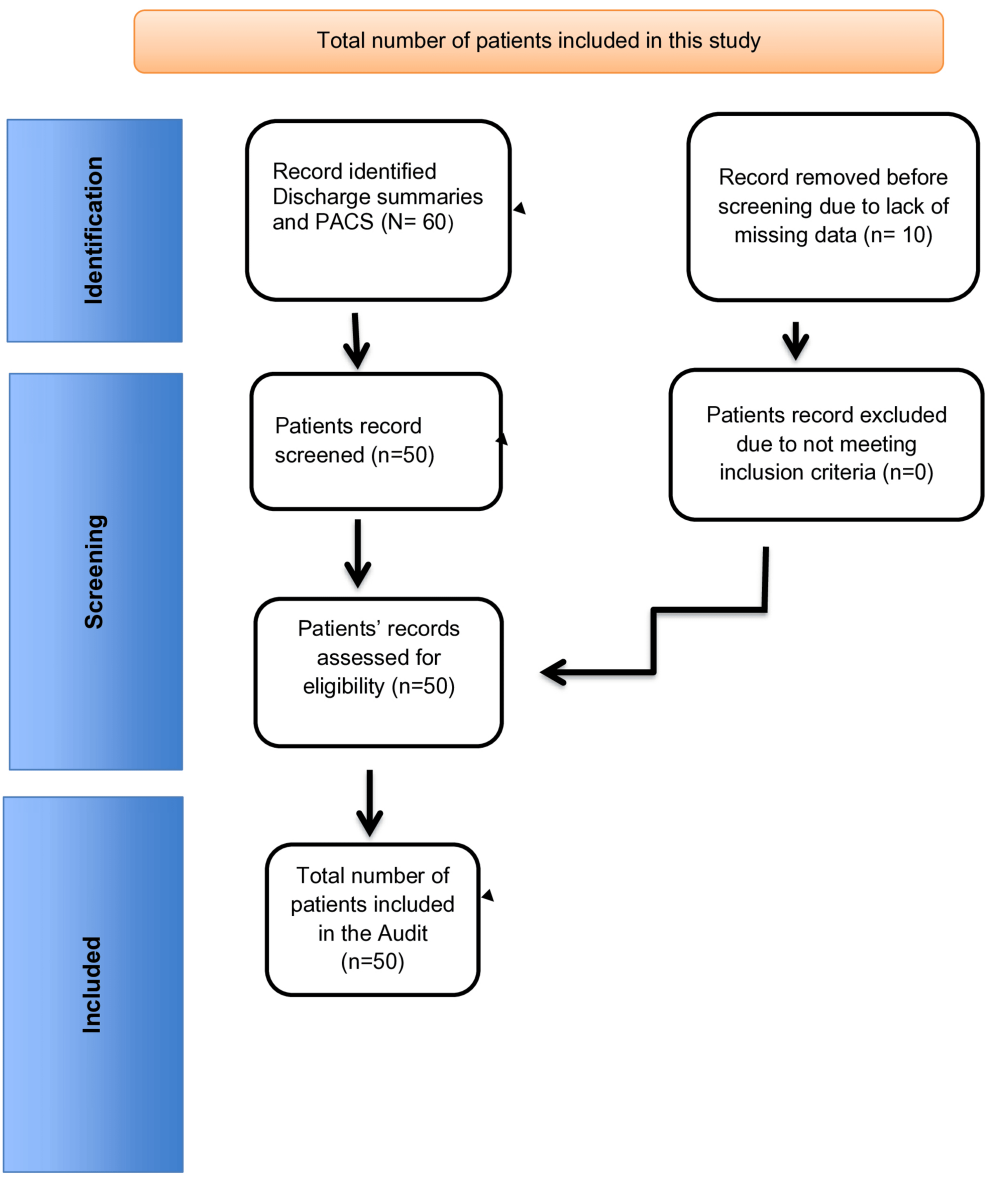
Flowchart showing the number of patients included in this study PACS: Picture Archiving and Communication System

This retrospective audit evaluates the investigation and management of thyroid nodules in clinical practice at a district general hospital in London, comparing it with the 2014 BTA guidelines. The audit focused on including U-scores in US reports and the appropriateness of FNAC based on the U-score classification. The audit included all thyroid and/or neck US reports performed within the last 12 months from January 1, 2023, to December 31, 2023, identifying at least one thyroid nodule. Nonthyroid US reports were excluded. All patients' identifiable data were coded and deidentified. Data search for identifying patients with thyroid nodules was done by using diagnostic codes in the discharge summaries and by using the Picture Archiving and Communication System (PACS) system over the past 12 months. The data search was conducted by four researchers over a month's time. Any disagreements were resolved through mutual consensus and with input from the most senior researcher. Patients who had undergone nonthyroid US scans and patients with missing data were excluded from the study. A total of 10 patients were excluded from this study due to missing data despite our best efforts to retrieve the data, as shown in the flowchart (Figure 1). The audit was registered with our local audit and research department under the registration number: L-209-22.

Inclusion criteria

Patients with US reports identifying at least one thyroid nodule within the last 12 months and reports including a U-score (U1-U5) or an alternative classification system, such as Thyroid Imaging Reporting and Data System (TI-RADS), were included.

Exclusion criteria

The US reports that did not identify or classify patients with nonthyroid US scans, those with thyroid nodules, or patients with missing data were excluded.

Selection strategy

A total of 60 cases were reviewed to assess compliance with BTA 2014 guidelines. A search was conducted in the PACS to identify all thyroid and neck US reports performed within the past 12 months. The presence of a U-score (U1-U5) or an alternative classification system (e.g., TI-RADS) score was reviewed in each US report. If no U-score was provided, a retrospective U-score was assigned based on the images reviewed by a consultant radiologist. US reports were assessed to confirm whether FNAC was performed for the identified nodules and whether any reasons were provided for deviating from the guidelines. Cytology reports were reviewed to verify if FNAC was carried out and to assess whether the results correspond to the classification based on the U-score.

Aims and objectives

The aims and objectives were subdivided into three leading indicators. The primary aim was to evaluate the initial thyroid US report for inclusion of U-scores. The second aim was to evaluate whether FNAC confirmation of these nodules was done in line with the U-score classification. Thyroid nodules with U1-U2 do not require FNAC in the absence of any other high-risk features, whereas thyroid nodules with U3-U5 scores should have FNAC performed unless there is an adequate clinical reason not to [8]. Finally, it should be noted whether the clinical reason for not adhering to the guidelines was documented in patients' notes as part of good medical practice.

The quantitative analysis focused on determining the percentage of US reports that include a U-score and calculating the proportion of FNAC procedures performed per the U-score classification. Using a qualitative study, we examined and categorized the reasons for deviations from the guidelines. Finally, the compliance assessment was compared to the actual practices with the recommended BTA 2014 guidelines to determine the level of adherence. The audit ensured compliance with institutional ethical standards, maintaining patient confidentiality in line with data protection regulations. No direct patient interaction is required as the study relies on retrospective data.

## Results

**Figure 2:**
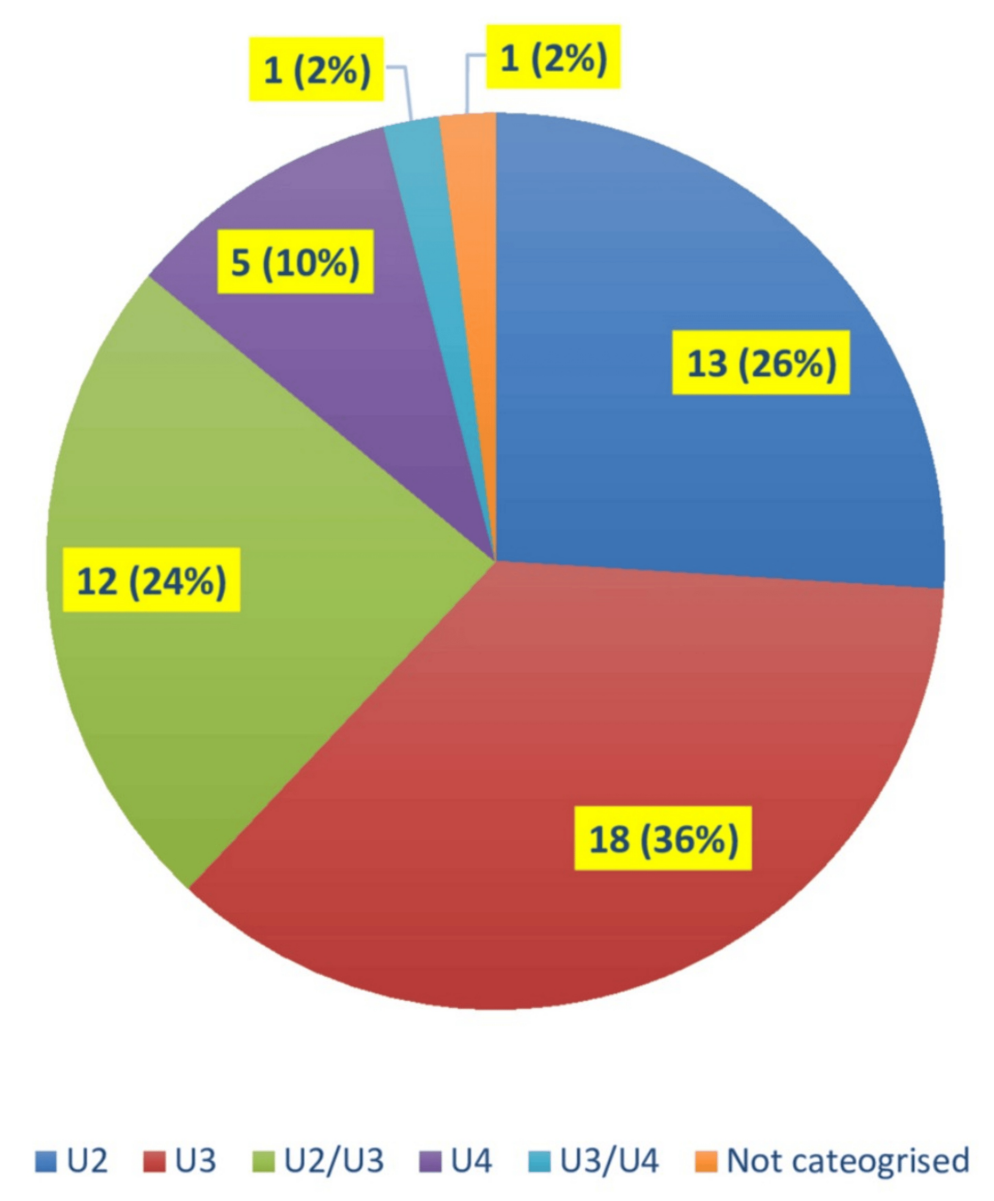
Pie chart showing the frequency of each U-score category cases

The audit reviewed 50 cases. Of these, 49 had a US report that included a U-score classification, while one did not. The distribution of cases across U-score categories was as follows: 13 cases were classified as U2, 18 cases as U3, 12 cases as U2/U3, five cases as U4, one case as U3/U4, and one case did not have any U-score categorization (Figure 2).

**Figure 3:**
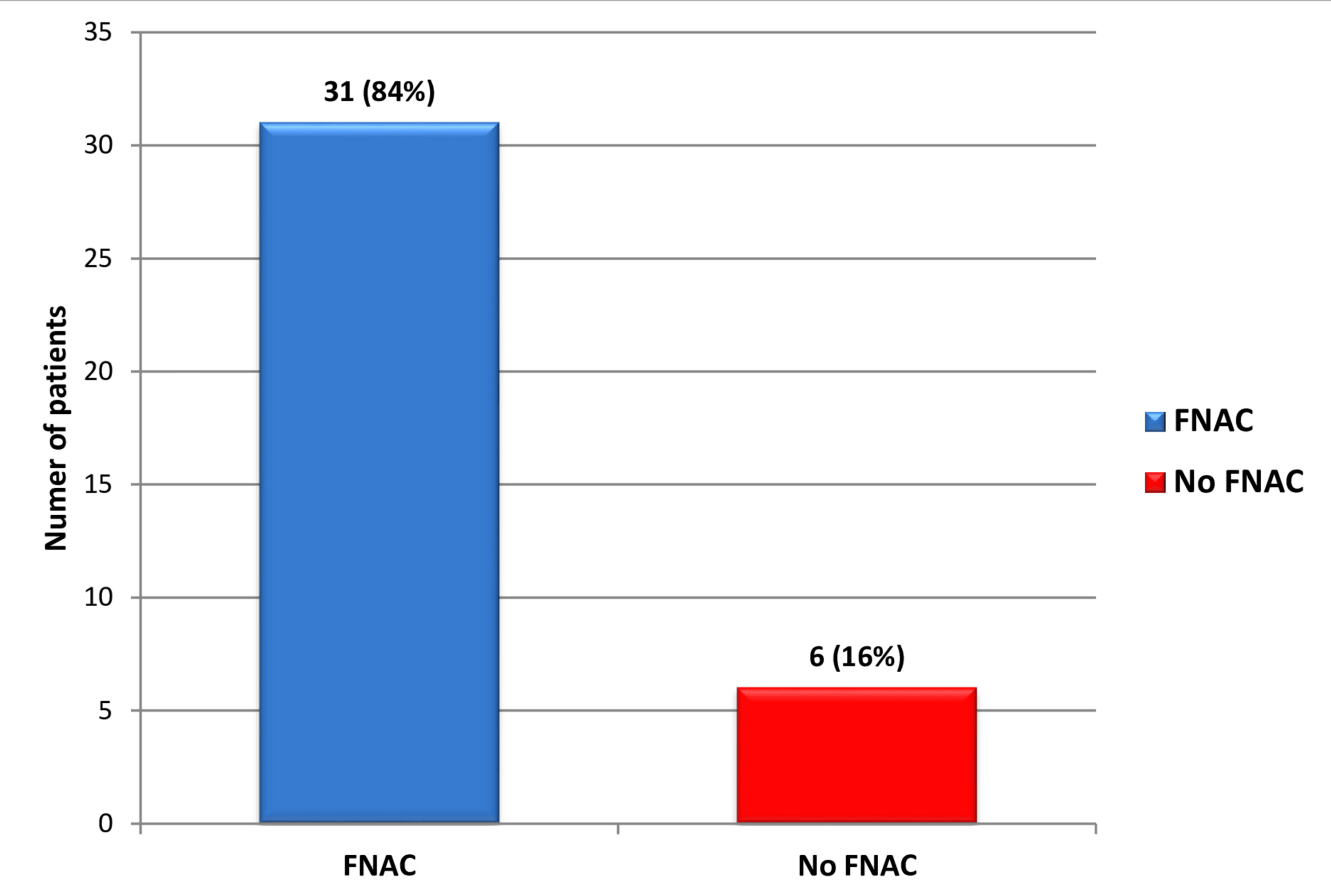
Pie chart showing the percentage of patients with and without FNAC FNAC: fine needle aspiration cytology

Regarding the necessity of FNAC based on US findings, 13 cases did not require FNAC, while 37 cases met the criteria for FNAC based on their U-score classification. Following the US, FNAC was performed in 31 cases, whereas six cases requiring FNAC did not proceed with the procedure, and no clear reason was provided for this (Figure 3).

**Figure 4:**
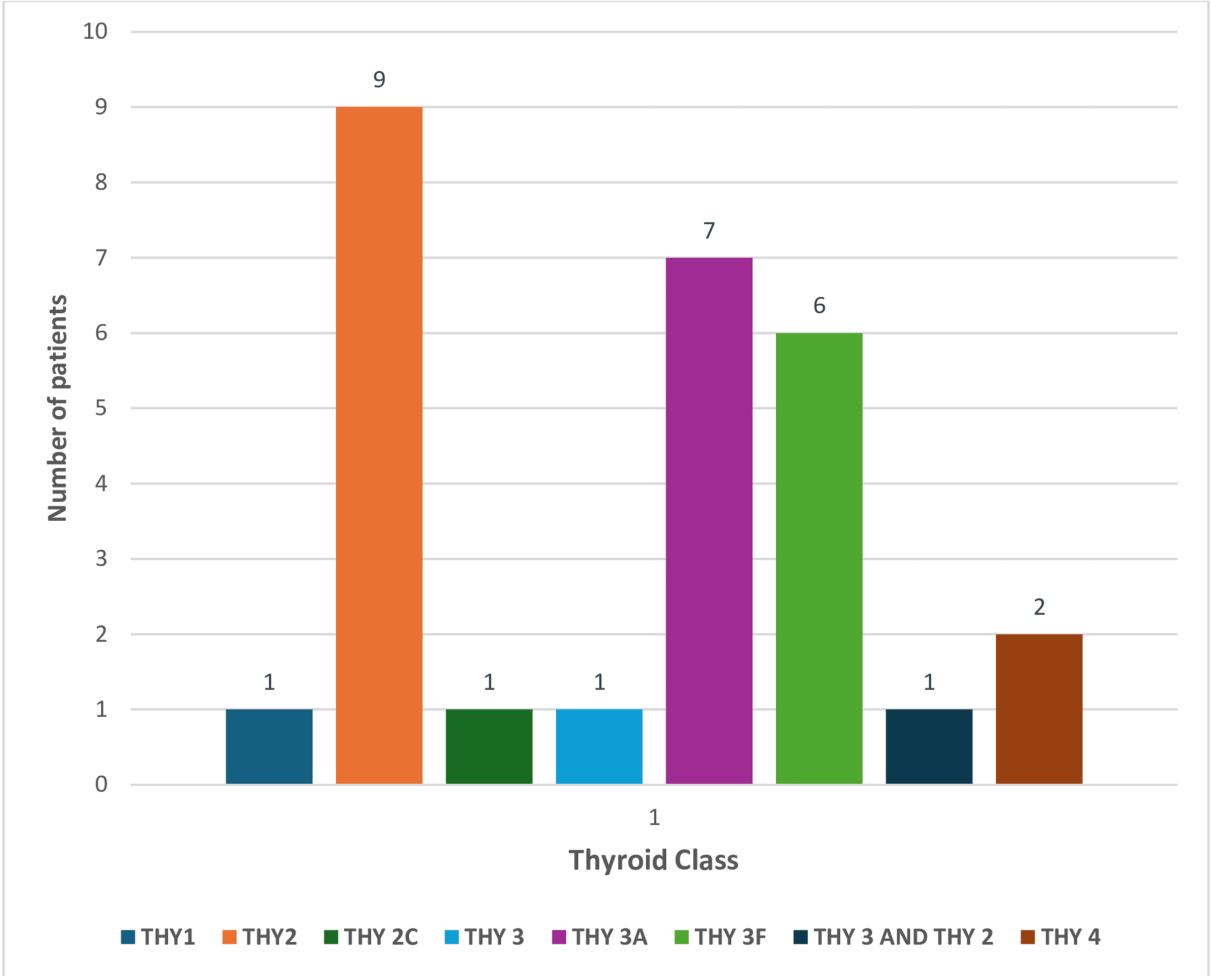
Bar chart showing the number of patients with THY classification as per the Royal College of Pathologists

A total of 28 cases were classified according to the THY classification system. Among them, one case was THY 1, nine were THY 2, one was THY 2c, one was THY 3, seven were THY 3A, six were THY 3F, one showed overlapping features of THY 3 and THY 2, and two cases were THY 4. This classification aids in risk assessment and guides further management of thyroid lesions (Table 1, Figure 4).

**Table 1:** The number of patients with THY classification as per the Royal College of Pathologists

Category	Total number of cases, including percentages
THY1	1
THY2	9
THY2C	1
THY3	1
THY3A	7
THY3F	6
THY3 and THY2	1
THY4	2

A total of 31 cases underwent FNAC. Of these, 28 were successfully classified according to the THY classification system. One case was not reported, and two showed THY 3 cytology but could not be further subclassified into THY 3A or THY 3F. FNAC could not be performed in one additional case due to increased vascularity. Among all 31 cases assessed, five were confirmed to have malignancy. Out of these, four underwent hemithyroidectomy, and one had local excision. Additionally, three patients underwent hemithyroidectomy with histology revealing benign adenomas, most commonly follicular adenomas.

Compliance with guidelines

**Figure 5:**
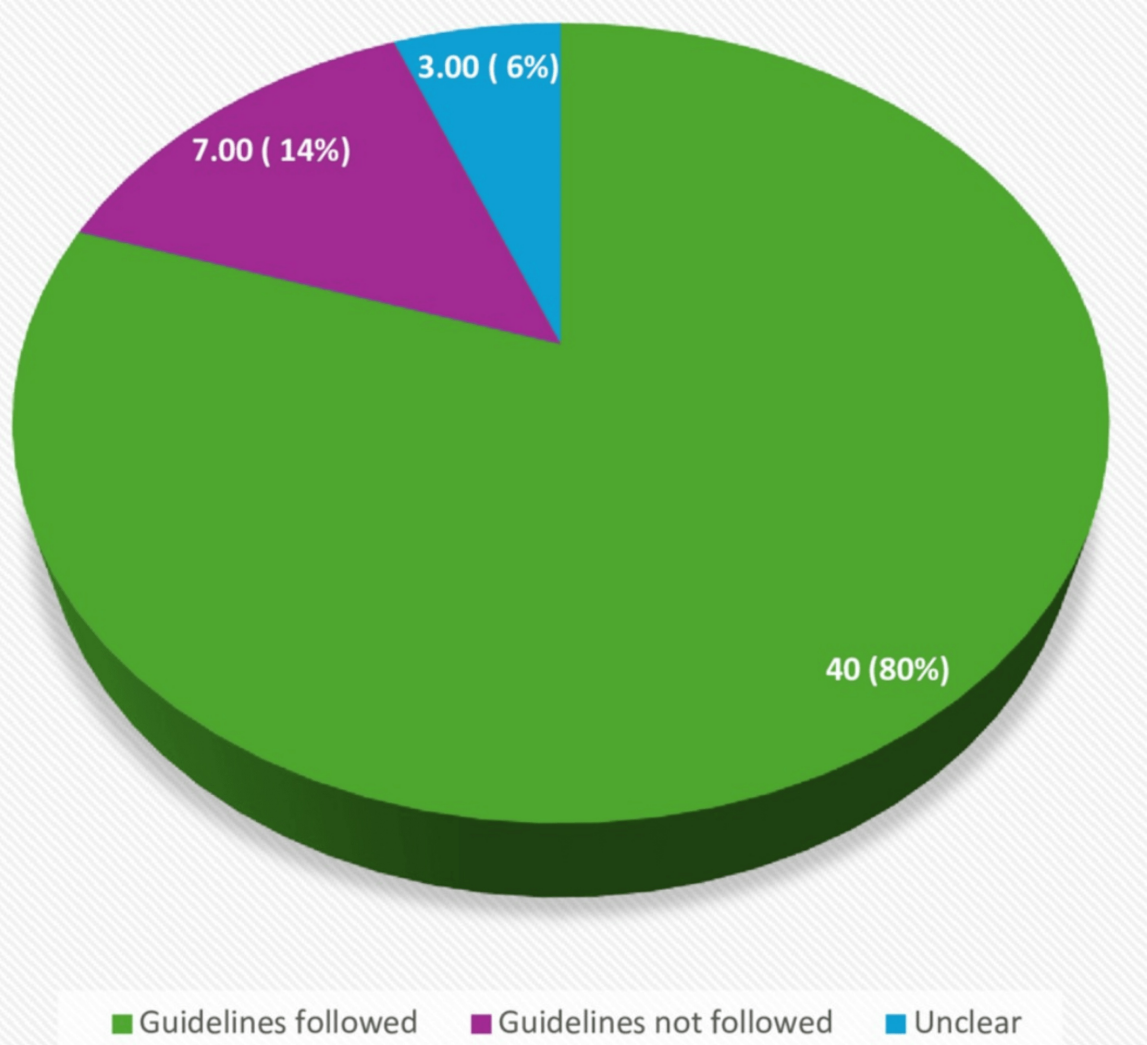
Pie chart showing the percentage of cases following guidelines (all U-score categories)

Overall, 80% of cases adhered to the BTA 2014 guidelines, 14% did not follow the recommended guidelines, and 6% had unclear adherence to the guidelines (Figure 5).

For individual U-score categories

BTA 2014 guidelines were followed in all 13 (100%) cases with U2 nodules, and guidelines to have fine needle cytology were not followed in one case in the U3 nodule group despite meeting the criteria. The only case with a U3/U4 classification followed the guidelines, and all five (100%) cases with U4 nodules adhered to the guidelines.

**Figure 6:**
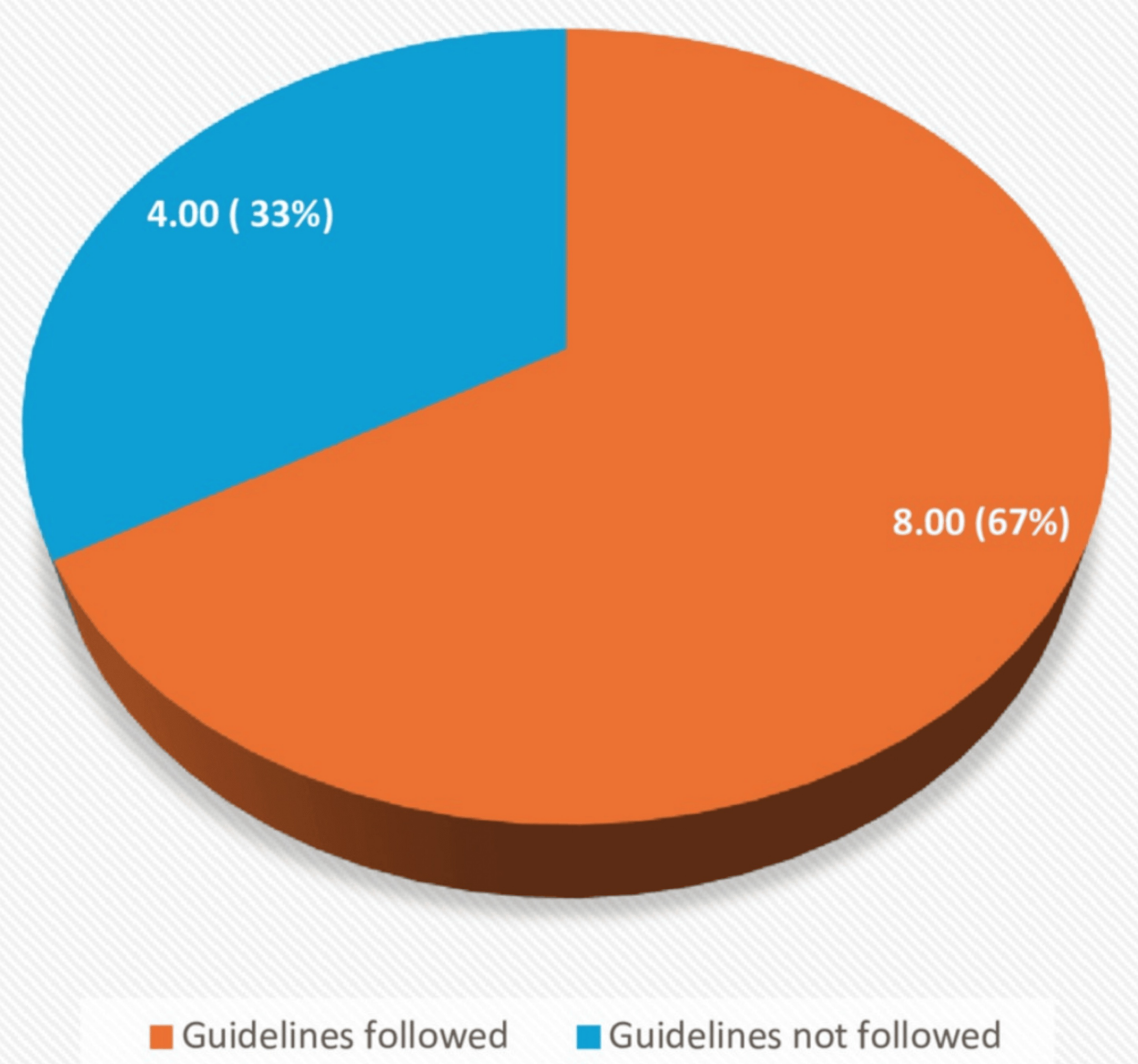
Pie chart showing the percentage of cases following guidelines (all U2/U3 categories)

For U2/U3 category cases, 12 were identified, of which eight followed the guidelines, while four did not. Among these four cases, the reasons for guideline deviation included the demise of one patient, the second one later got diagnosed with receptor-positive Graves' disease, the third one underwent a repeat neck US, and the fourth patient was an elderly patient refusing further management (Figure 6).

Malignancy detection

FNAC was performed in 35 cases, and malignancy was identified in five cases. Four patients underwent hemithyroidectomy, while one had a local excision. Additionally, three patients who had hemithyroidectomy were found to have benign adenomas, with follicular adenoma being the most common diagnosis.

## Discussion

The retrospective study findings demonstrate high compliance with the BTA 2014 U-classification system, with 98% of US reports including a U-score. This aligns well with the BTA recommendation that all thyroid US should incorporate a U-score to guide the need for FNAC. Regarding FNAC adherence, 80% of cases followed the guidelines, which is a reasonable compliance rate but leaves room for improvement. The breakdown of cases shows that all U2 and U4 nodules were managed correctly, consistent with BTA guidelines stating that U2 nodules do not require FNAC while U4 nodules should undergo FNAC. However, one patient with a U3 nodule did not receive FNAC, representing a minor deviation from the recommended protocol. Similarly, for U2/U3 cases, deviations were noted in four cases, although clinical justifications, such as comorbidities or alternative diagnoses, were identified.

Thyroid nodules are mainly detected as incidental findings during imaging studies for other reasons, or occasionally during physical examination [2,3]. Most thyroid nodules are benign and asymptomatic and do not require any intervention. Malignant nodules, on the contrary, require surgical excision [3]. US scan is the preferred imaging modality for assessing thyroid nodules. The autopsy data indicate that about 50% of thyroid nodules are greater than 1 cm in patients without any clinical evidence of thyroid disease, whereas the prevalence of palpable thyroid nodules is approximately 4%-7% [3,9,10]. Thyroid nodular disease is common in the United Kingdom and the United States, with the prevalence of palpable nodules in approximately 5% of women [9]. The prevalence of clinically apparent thyroid nodules in the Framingham study was 6.4% and 1.5% in women and men, respectively [11]. Given the significant difference between palpable thyroid nodules and postmortem findings of undetected thyroid nodules, the actual prevalence is higher than previously considered. Imaging has significantly increased the detection of the nonpalpable thyroid nodules with a detection rate of 20% on US scans, 25% on contrast-enhanced chest computerized tomography scans of the thorax, 15% on magnetic resonance imaging of the neck, and 30% on positron-emission tomography-computed tomography (PET-CT) scans [10]. Most thyroid nodules detected as incidental findings can be managed by the same principle of history and examination of palpable lumps, except those nodules detected on CT or US scan and less than 1 cm in size, and avid lesions detected on PET-CT. The former do not require any further investigations, whereas the latter need urgent referral to rule out malignancy [11].

Solitary thyroid nodules are more likely to be cancerous than single nodules within a multinodular gland, with the incidence of malignancy ranging from 2.7%-30% and 1.4%-10% for the former and latter, respectively [12]. US scan is useful in characterizing nodular features associated with malignancy, such as hypoechogenicity, microcalcifications, irregular margins, increased vascularity, and the absence of a halo, which are features suggestive of malignancy [13,14]. Due to the subjectivity of the US findings, histological confirmation is necessary in differentiating between benign and malignant thyroid tumors. Similarly, radioisotope scans provide useful information about the functioning of thyroid nodules but provide limited information about their size [14]. Approximately 80%-85% of thyroid nodules are cold, with only 10% of these nodules representing a malignancy [1,14]. On the contrary, the hot nodules account for 5% of all nodules, and the risk of malignancy is <1% for these nodules. CT and magnetic resonance imaging scans are not indicated for the initial evaluation of thyroid nodules, although both have 100% sensitivity for evaluating the extent of large substernal goiters [1].

 A total of 2,654 new cases and 346 deaths of thyroid cancer were reported in the UK in 2010, based on the data from 2017 to 2019. This equals 11 new cases every day or 4,000 new cases of thyroid cancer every year in the country [2,14]. Still, such malignancy accounts for 1% of all new cancers in the UK, and it is the 15th most common among women and 20th most frequent among men [14]. The one-year survival rate for thyroid cancer in England is 84.3% based on data from 2013 to 2017, and the survival rate is higher in women than in men [14]. Furthermore, 99.2% of patients diagnosed with thyroid cancer aged 15-44 have a 10-year survival rate, compared to 57.7% for those aged 75-99.

Another group of patients for whom there is a lack of data is pregnant and postpartum women. Several studies have examined the prevalence of thyroid nodules in pregnant women using US scans, with a reported prevalence ranging from 3% to 30% [15-17]. Studies have shown that the size of thyroid nodules doubles during pregnancy, and about a fifth of women with one thyroid nodule during the first trimester also develop a second nodule toward the end of pregnancy [16-18]. Three cross-sectional studies reported the prevalence of malignant thyroid nodules during pregnancy to vary from 12%-43% [19-21].

In terms of management, most primary thyroid carcinomas require surgical resection by either lobectomy or total thyroidectomy, whereas metastatic tumors and lymphomas are mostly treated with chemotherapy and/or radiation. Thyroid tumors with high-risk features such as large size greater than 4 cm, extrathyroidal extension, bilateral nodularity, neck lymph node involvement, history of neck radiation, and distant metastasis warrant total thyroidectomy. These patients may also require removal of neck lymph nodes. On the other hand, localized parathyroid cancers less than 4 cm in size may be treated with partial lobectomy or total thyroidectomy, whereas active surveillance is recommended for patients with thyroid microcarcinomas (<1 cm) without any metastasis or adverse features [4,5,15].

Limitations

The main limitations of this study include its retrospective nature and small sample size. Additionally, this is a single-centered study, so results may not be generalizable to other centers. There is also a small risk of documentation bias in retrospective studies.

Recommendations

Two key recommendations are proposed to improve adherence to the BTA 2014 guidelines and ensure standardized reporting of thyroid nodules. First, all US rooms should display laminated educational U-scoring guidelines with corresponding US examples. This will serve as a quick reference guide for radiologists and sonographers, reinforcing the correct classification of thyroid nodules. By providing clear visual examples of U1 to U5 categories, this resource will aid in accurate and consistent U-score assignment, reducing variability in reporting. Ensuring that all US reports include a U-score will facilitate appropriate decision-making regarding FNAC and improve patient management.

Second, regular teaching sessions should be conducted for all staff involved in thyroid US and FNAC procedures. This session should cover the importance of standardized U-scoring, guideline-based FNAC recommendations, and common pitfalls in thyroid nodule assessment. By enhancing awareness and knowledge among radiologists, sonographers, and clinicians, the training will help improve compliance with established guidelines, reduce unnecessary FNACs, and ensure that FNAC is performed if clinically indicated. These recommendations will improve guideline adherence and diagnostic accuracy and optimize patient care in managing thyroid nodules. Additionally, a dedicated clinic to follow these patients would be useful to minimize the loss of follow-ups.

## Conclusions

Our retrospective study confirms that the system effectively guides FNAC decisions in most cases, compared to the expected performance of BTA U-classification. However, real-world implementation shows occasional deviations due to clinical discretion, patient preference, or logistical factors. These findings suggest that while the BTA U-classification is a robust framework for thyroid nodule management, continued efforts in education and adherence monitoring are necessary to achieve full compliance. Thyroid nodule management should be streamlined through a one-stop clinic to avoid unnecessary delays and work duplication.
